# Dietary frequency, body satisfaction, anxiety symptoms, and depressive symptoms among offspring of healthcare workers in China

**DOI:** 10.3389/fnut.2026.1908874

**Published:** 2026-10-15

**Authors:** Tingke Xu, Rongxiu Ding, Chao Lin, Qiuhua Zhang, Pan Ding

**Affiliations:** 1 Department of Health Information, Sir Run Run Shaw Hospital, Zhejiang University School of Medicine, Hangzhou, Zhejiang, China; 2 School and Hospital of Stomatology, Wenzhou Medical University, Wenzhou, Zhejiang, China; 3 Department of Clinical Laboratory, Key Laboratory of Clinical Laboratory Diagnosis and Translational Research of Zhejiang Province, The First Affiliated Hospital of Wenzhou Medical University, Wenzhou, Zhejiang, China; 4 School of Public Health, Wenzhou Medical University, Wenzhou, Zhejiang, China

**Keywords:** anxiety symptoms, body satisfaction, depressive symptoms, dietary frequency, healthcare workers’ offspring

## Abstract

**Background:**

Dietary behaviors may be associated with body image and emotional health in young people, but evidence integrating these outcomes in Chinese populations remains limited. This study examined associations of dietary frequency with body satisfaction, anxiety symptoms, and depressive symptoms.

**Methods:**

This cross-sectional study included 584 offspring of healthcare workers aged 10–24 years in Zhejiang, China. A 10-item food-frequency questionnaire generated a frequency-based score reflecting more frequent consumption of meat, fish, vegetables, fruit, eggs, soy products, and dairy products and less frequent consumption of pickled, sugary, and fried foods. Multivariable logistic regression and restricted cubic splines were used to assess associations and dose–response patterns. Counterfactual mediation analysis estimated indirect associations through body satisfaction.

**Results:**

The proportions screening positive for anxiety and depressive symptoms were 23.1 and 33.6%, respectively. In fully adjusted models, higher dietary frequency scores were associated with higher odds of body satisfaction (OR = 1.21, 95% CI: 1.02–1.44) and lower odds of anxiety (OR = 0.65, 95% CI: 0.52–0.79) and depressive symptoms (OR = 0.84, 95% CI: 0.70–0.99). Restricted cubic spline analyses indicated approximately linear associations, with no evidence of nonlinearity. For each 1-SD increase in the dietary frequency score, indirect associations through higher body satisfaction were observed for anxiety (OR = 0.97, 95% CI: 0.93–0.99; 6.8%) and depressive symptoms (OR = 0.96, 95% CI: 0.92–0.99; 17.3%).

**Conclusion:**

Higher dietary frequency scores were associated with greater body satisfaction and lower odds of anxiety and depressive symptoms among healthcare workers’ offspring. These findings cannot be generalized to other young people in China.

## 1 Introduction

Adolescence and young adulthood are sensitive developmental periods characterized by rapid physical growth, identity formation, increasing academic and social demands, greater autonomy over daily behaviors, and heightened emotional vulnerability (1). The World Health Organization (WHO) defines young people as individuals aged 10–24 years, encompassing adolescents aged 10–19 years and youth aged 15–24 years (2). WHO estimates that approximately one in seven (14.3%) adolescents aged 10–19 years experiences a mental disorder (3), with anxiety and depressive disorders among the leading causes of illness and disability in this age group. This burden is also substantial in China. A large-scale study of Chinese adolescents reported depressive and anxiety symptoms in 25.6 and 26.9% of participants, respectively (4). A pandemic-period meta-analysis restricted to participants younger than 18 years yielded pooled estimates of 22% for depressive symptoms and 25% for anxiety symptoms (5). Beyond anxiety and depressive symptoms, body satisfaction is another important psychological concern during adolescence and early adulthood. Longitudinal studies have shown that body dissatisfaction is associated with later depressive symptoms and anxiety trajectories, suggesting that body-related self-evaluation is closely linked to youth mental health (6, 7). Together, the substantial burden of emotional symptoms and the close relationship between body-related evaluation and psychological well-being highlight the need to identify modifiable behavioral factors relevant to both body satisfaction and emotional health among young people.

Diet is a potentially modifiable behavior relevant to young people’s psychological health. China’s nutrition transition has led traditional plant-rich diets to coexist with increasing consumption of animal-source, energy-dense, and highly processed foods (8, 9), while young people’s food choices become increasingly self-directed with growing autonomy. Although healthier dietary patterns have generally been associated with better mental health in children and adolescents (10), findings vary according to the dietary exposure and psychological outcome examined. For example, a longitudinal study of Canadian adolescents found that sugar-sweetened beverage consumption predicted more severe anxiety and depressive symptoms, whereas fruit and vegetable consumption was associated with psychological well-being but not with anxiety or depressive symptoms (11). Dietary behavior is also intertwined with body satisfaction: body-related concerns may influence food choices and weight-control practices, while habitual dietary behaviors may be associated with physical development, body evaluation, and emotional well-being (12). Because body satisfaction is prospectively associated with depressive and anxiety symptoms (6, 7), it may constitute a relevant psychosocial dimension of diet–emotional health associations. However, previous studies have generally examined emotional symptoms and body image separately and have focused largely on individual foods or culturally specific dietary patterns (10–12). Consequently, evidence remains limited regarding the independent associations of an overall dietary-frequency score with body satisfaction, anxiety symptoms, and depressive symptoms within a single analytical framework, while the potential mediating role of body satisfaction remains underexplored among Chinese young people.

Offspring of healthcare workers may experience a distinctive family context shaped by parental work–family conflict and health-related practices, which may influence emotional well-being and eating behaviors (13, 14). However, research in this population has focused mainly on pandemic-related mental health, with little attention to diet, body satisfaction, and emotional symptoms within a single framework (15). Using data from a cross-sectional survey of offspring of healthcare workers in Zhejiang Province, China, this study aimed to examine the associations of the dietary frequency score with body satisfaction, anxiety symptoms, and depressive symptoms. Specifically, we sought to (1) assess the associations of the dietary frequency score with body satisfaction, anxiety symptoms, and depressive symptoms; (2) characterize the dose–response relationships between the dietary frequency score and these psychological outcomes; (3) determine whether these associations differed across demographic and lifestyle subgroups; and (4) evaluate whether body satisfaction potentially mediated the associations between the dietary frequency score and anxiety and depressive symptoms.

## 2 Methods

### 2.1 Study design and participants

This cross-sectional survey of offspring of healthcare workers was conducted between July and September 2025 through four hospitals in Zhejiang Province, China. The questionnaire was jointly developed by six experts in public health, psychology, and sociology, with reference to assessment instruments used in previous large-scale cohort studies (16, 17). It collected information on demographic characteristics, dietary behaviors, lifestyle factors, and psychological health-related variables. Sample size was estimated using the formula for estimating a population proportion in a cross-sectional study, $n=Z_{\alpha /2}^{2}P(1-P)/d^{2}$ . Previous studies (18, 19) have reported anxiety and depressive symptom rates ranging from approximately 19.85–34.70% among Chinese young people. Assuming a two-sided *α* of 0.05 and an absolute precision of 5%, the estimated minimum sample size ranged from 245 to 349 participants. After allowing for a 10% non-response or invalid-response rate, the maximum required sample size was estimated to be 388 participants.

Using convenience sampling, offspring of healthcare workers were recruited and surveyed face to face. Before questionnaire administration, participants were informed of the study background, objectives, confidentiality procedures, and their right to withdraw at any time without adverse consequences. Across the four participating hospitals, 609 offspring of healthcare workers were initially approached. Of these, 487 were approached through the First Affiliated Hospital of Wenzhou Medical University and 49 through Sir Run Run Shaw Hospital. An additional 37 were approached through the School and Hospital of Stomatology of Wenzhou Medical University and 36 through Wenzhou People’s Hospital. One individual did not complete the questionnaire, leaving 608 returned questionnaires. In accordance with the World Health Organization definition of young people as those aged 10–24 years (2), we excluded 24 respondents outside this age range. The final analytic sample therefore comprised 584 participants (Supplementary Figure S1). Participants aged 18 years or older provided written informed consent. For participants younger than 18 years, both the minor and a parent or legal guardian agreed to participation and signed the written informed consent form. The study was conducted in accordance with the Declaration of Helsinki and was approved by the Institutional Research Ethics Committee of Wenzhou Medical University (YJ-2024-09-01).

### 2.2 Assessment of dietary frequency

Dietary consumption frequency over the preceding month was assessed using a 10-item food-frequency questionnaire covering meat, fish, vegetables, fruit, eggs, soy products, dairy products, pickled foods, sugary foods and beverages, and fried foods. The questionnaire was adapted from dietary assessment items used in the Chinese Longitudinal Healthy Longevity Survey (CLHLS) (20, 21) and the China Family Panel Studies (CFPS) (22), whose dietary assessment approaches have shown acceptable reproducibility and validity in Chinese populations. For each food group, response options were rarely or never, 1–2 times/week, 3–5 times/week, and almost every day, coded as 1, 2, 3, and 4, respectively. Scores for pickled foods, sugary foods/drinks, and fried foods were reverse-coded. The scores for the 10 items were summed to yield a dietary frequency score ranging from 10 to 40, with higher scores indicating more frequent consumption of the seven positively scored food categories and less frequent consumption of pickled, sugary, and fried foods.

### 2.3 Assessment of body satisfaction

Body satisfaction was assessed using the six-item Body Image States Scale (BISS), which assesses individuals’ momentary evaluative and affective experiences regarding their body and physical appearance (23, 24). Each item was rated on a nine-point bipolar response scale, with item-specific anchors reflecting satisfaction with physical appearance, body size and shape, weight, physical attractiveness, and evaluations of appearance relative to usual feelings or others (25). Items 2, 4, and 6 were reverse-scored so that higher scores consistently indicated greater body satisfaction. The six item scores were summed to yield a total score ranging from 6 to 54, with higher scores indicating greater body satisfaction. In the present sample, the BISS demonstrated excellent internal consistency (Cronbach’s *α* = 0.965). The BISS has also been applied in Chinese school-age and early-adolescent populations (24, 26) as well as university students (25), with previous studies reporting satisfactory internal consistency and factor-structural performance. For the primary analyses, participants were categorized according to the sample median BISS score (31 points), with scores ≥31 classified as higher body satisfaction and scores <31 as lower body satisfaction.

### 2.4 Assessment of anxiety symptoms

Anxiety symptoms were measured using the Generalized Anxiety Disorder 7-item scale (GAD-7) (27). The GAD-7 assesses the frequency of seven anxiety-related symptoms during the preceding 2 weeks: feeling nervous, anxious, or on edge; being unable to stop or control worrying; worrying too much about different things; trouble relaxing; being so restless that it is hard to sit still; becoming easily annoyed or irritable; and feeling afraid as if something awful might happen. For each item, participants responded on a four-point response scale ranging from “not at all” to “nearly every day,” with scores assigned from 0 to 3, respectively (28). The total score ranges from 0 to 21, with higher scores indicating greater severity of anxiety symptoms. In this study, the GAD-7 showed excellent reliability, with a Cronbach’s *α* of 0.962. The GAD-7 has been shown to be a valid and practical tool for screening anxiety symptoms among Chinese adolescents and young adults (29). Consistent with previous studies, a GAD-7 score ≥7 was used to define the presence of anxiety symptoms (30).

### 2.5 Assessment of depressive symptoms

Depressive symptoms were assessed using the conventional two-item Patient Health Questionnaire (PHQ-2), which assesses anhedonia and depressed mood over the preceding 2 weeks. Each item is rated from 0 (“not at all”) to 3 (“nearly every day”), yielding a total score of 0–6, with higher scores indicating a greater depressive symptom burden (31). Owing to its brevity and acceptable screening performance relative to the PHQ-9, the PHQ-2 has been widely applied in population-based and epidemiological research (32). Evidence from previous studies also supports its utility for identifying depressive symptoms among adolescents and young adults in different cultural settings (33–35). In the present study, a total score ≥2 was used to identify depressive symptoms (36, 37).

### 2.6 Assessment of covariates

Covariates were selected *a priori* based on previous research on dietary intake, lifestyle behaviors, and mental health among young people (11). Demographic covariates included sex (male, female), age (continuous), educational stage (senior high school or below, university), place of residence (city, town, rural), parental education (junior high school or below, senior/vocational school, college or above), and family economic status (affluent, well-off, adequate, poor). Lifestyle-related covariates included sleep duration (continuous), social media time (continuous), sedentary time (continuous), physical activity time (continuous), and body type (underweight, normal weight, overweight, obesity). Family-related behavioral covariates were each assessed using a single item: parental healthy lifestyle habits (always, often, sometimes, never) and parental attention to dietary balance (attentive, moderately attentive, indifferent).

Parental education was defined according to the higher educational attainment of either parent. Physical activity time, sedentary time, and social media time were self-reported by participants based on their average daily duration (38). Physical activity time referred to daily time spent in physical activities (e.g., running, swimming, or team sports), while sedentary time represented daily time spent in sedentary behaviors excluding sleep (e.g., studying and screen use). Social media time was defined as the average daily time spent on social media platforms such as WeChat, Douyin, and Kuaishou. Body mass index (BMI) was calculated from self-reported weight and height as weight in kilograms divided by height in meters squared (kg/m^2^) (39). Body type was classified using age-appropriate criteria. For participants aged 10–17 years, the age- and sex-specific WHO 2007 BMI-for-age reference (40) was used to classify underweight (<−2 SD), normal weight (−2 SD to +1 SD), overweight (> + 1 SD to +2 SD), and obesity (> + 2 SD). For participants aged ≥18 years, Chinese adult BMI criteria were applied (41): underweight (<18.5 kg/m^2^), normal weight (18.5–23.9 kg/m^2^), overweight (24.0–27.9 kg/m^2^), and obesity (≥28.0 kg/m^2^). Complete data were available for all variables included in the analyses.

### 2.7 Statistical analyses

Continuous variables were summarized as means and standard deviations (SDs), and categorical variables as frequencies and percentages. Differences in participant characteristics between males and females were assessed using Student’s t-test for continuous variables and the chi-square test for categorical variables.

The dietary frequency score was standardized before regression analyses, and odds ratios were estimated per 1-SD increase in the score. Binary logistic regression models were used to examine the associations of the dietary frequency score with higher body satisfaction, anxiety symptoms, and depressive symptoms. Odds ratios (ORs) and 95% confidence intervals (CIs) were reported. Model I adjusted for sex, age, educational stage, place of residence, parental education, and family economic status. Model II further adjusted for sleep duration, social media time, sedentary time, physical activity time, parental healthy lifestyle habits, parental attention to dietary balance, and body type. Multicollinearity among variables included in Model II was assessed using variance inflation factors (VIFs) and generalized variance inflation factors (GVIFs) for multi-category variables. No substantial multicollinearity was observed (Supplementary Table S1).

Restricted cubic spline (RCS) logistic regression was further used to examine potential nonlinear associations between the dietary frequency score and each outcome. Three knots were placed at the 10th, 50th, and 90th percentiles of the dietary frequency score distribution, corresponding to scores of 25, 29, and 34, respectively. The median score of 29 was used as the reference value. RCS models were adjusted for the same covariates as Model II. *p* values for the overall and nonlinear associations were calculated, and adjusted ORs with 95% CIs were plotted together with the distribution of the dietary frequency score.

Exploratory stratified analyses were conducted by age, sex, social media time, sleep duration, physical activity time, sedentary time, and body type. Multiplicative interaction terms between the dietary frequency score and each stratification variable were added to the regression models, and the corresponding interaction *p* values were used to assess effect modification. The *p* for interaction values were further adjusted using the Benjamini–Hochberg false discovery rate (FDR) procedure.

Counterfactual mediation analyses were conducted to examine whether higher body satisfaction statistically mediated the associations of the dietary frequency score with anxiety symptoms and depressive symptoms. For these analyses, the dietary frequency score was standardized to a *z* score (mean = 0, SD = 1), so that effect estimates represented a 1-SD increase in the dietary frequency score. Higher body satisfaction was modeled as a binary mediator using logistic regression, and anxiety and depressive symptoms were modeled as binary outcomes using logistic regression. Regression models were fitted using the R *stats* package, and natural effects were estimated using parametric g-computation. The pure natural direct effect (PNDE), total natural indirect effect (TNIE), and total effect (TE) were reported as ORs with 95% CIs. Percentile-based 95% CIs were obtained from 5,000 bootstrap resamples using the R *boot* package. Mediation proportions were also estimated.

To assess the robustness of the findings, several sensitivity analyses were conducted. First, the analyses were repeated among participants recruited from the First Affiliated Hospital. Second, each of the 10 food-frequency items was examined separately for the three outcomes. Finally, reverse-direction mediation analyses were performed with anxiety or depressive symptoms as the exposure, higher body satisfaction as the mediator, and the standardized dietary frequency score as the outcome, with 95% CIs obtained from 5,000 bootstrap resamples.

All statistical analyses were performed using R software version 4.5.2. A two-sided *p* < 0.05 was considered statistically significant.

## 3 Results

### 3.1 Basic characteristics of participants

Among the 584 participants included in the analysis, the mean age was 18.88 ± 3.00 years, 67.3% were female, 50.3% resided in cities, and 78.9% were university students. Compared with males, females were slightly older (19.08 ± 2.72 vs. 18.47 ± 3.48 years), were more likely to be university students (83.2% vs. 70.2%), and had a higher proportion of normal weight (65.6% vs. 55.5%) (all *p* < 0.05) (Table 1).

**Table 1:** Basic characteristics of participants by sex.

Characteristics	Overall (*n* = 584)	Male (*n* = 191)	Female (*n* = 393)	*p* value
Age, years, Mean ± SD	18.88 ± 3.00	18.47 ± 3.48	19.08 ± 2.72	**0.021**
Educational stage, *n* (%)				**<0.001**
Senior high school or below	123 (21.1)	57 (29.8)	66 (16.8)	
University	461 (78.9)	134 (70.2)	327 (83.2)	
Place of residence, *n* (%)				0.364
City	294 (50.3)	104 (54.5)	190 (48.3)	
Town	149 (25.5)	46 (24.1)	103 (26.2)	
Rural	141 (24.1)	41 (21.5)	100 (25.4)	
Parental education, *n* (%)				0.541
Junior high school or below	226 (38.7)	68 (35.6)	158 (40.2)	
Senior/vocational school	155 (26.5)	52 (27.2)	103 (26.2)	
College or above	203 (34.8)	71 (37.2)	132 (33.6)	
Family economic status, *n* (%)				0.196
Affluent	17 (2.9)	7 (3.7)	10 (2.5)	
Well-off	337 (57.7)	116 (60.7)	221 (56.2)	
Adequate	205 (35.1)	57 (29.8)	148 (37.7)	
Poor	25 (4.3)	11 (5.8)	14 (3.6)	
Sleep duration, h/day, Mean ± SD	8.70 ± 1.09	8.59 ± 1.05	8.76 ± 1.11	0.078
Social media time, h/day, Mean ± SD	5.33 ± 3.88	5.31 ± 4.47	5.35 ± 3.57	0.907
Sedentary time, h/day, Mean ± SD	4.84 ± 3.69	4.97 ± 3.99	4.77 ± 3.53	0.539
Physical activity time, h/day, Mean ± SD	2.57 ± 2.51	2.61 ± 2.19	2.54 ± 2.65	0.752
Parental healthy lifestyle habits, *n* (%)				0.312
Always	255 (43.7)	86 (45.0)	169 (43.0)	
Often	238 (40.8)	70 (36.6)	168 (42.7)	
Sometimes	68 (11.6)	28 (14.7)	40 (10.2)	
Never	23 (3.9)	7 (3.7)	16 (4.1)	
Parental attention to dietary balance, *n* (%)				0.690
Attentive	351 (60.1)	111 (58.1)	240 (61.1)	
Moderately attentive	199 (34.1)	67 (35.1)	132 (33.6)	
Indifferent	34 (5.8)	13 (6.8)	21 (5.3)	
Body type, *n* (%)				**<0.001**
Underweight	113 (19.3)	23 (12.0)	90 (22.9)	
Normal weight	364 (62.3)	106 (55.5)	258 (65.6)	
Overweight	74 (12.7)	42 (22.0)	32 (8.1)	
Obesity	33 (5.7)	20 (10.5)	13 (3.3)	

Student’s *t*-test was used to compare continuous variables, and the Chi-square test was used to compare categorical variables. Bold values indicated statistical significance *p* < 0.05. SD: standard deviation.

### 3.2 Associations of dietary frequency score with body satisfaction, anxiety symptoms, and depressive symptoms

Higher body satisfaction was identified in 305 (52.2%) participants, while 135 (23.1%) had anxiety symptoms and 196 (33.6%) had depressive symptoms. In the crude models, per 1-SD increase in the dietary frequency score, the odds of sample-defined higher body satisfaction increased by 25% (OR = 1.25, 95% CI: 1.06–1.47), whereas the odds of anxiety and depressive symptoms decreased by 37% (OR = 0.63, 95% CI: 0.52–0.78) and 21% (OR = 0.79, 95% CI: 0.67–0.95), respectively. These associations remained after covariate adjustment. Per 1-SD increase in the dietary frequency score, the odds of sample-defined higher body satisfaction increased by 21% (OR = 1.21, 95% CI: 1.02–1.44), whereas the odds of anxiety and depressive symptoms decreased by 35% (OR = 0.65, 95% CI: 0.52–0.79) and 16% (OR = 0.84, 95% CI: 0.70–0.99), respectively (Table 2).

**Table 2:** Associations of dietary frequency score with body satisfaction, anxiety symptoms, and depressive symptoms.

Outcomes	*n* (%)	Dietary frequency score, *OR* (95% CI)		
		Crude model	Model I	Model II
Higher body satisfaction	305 (52.2)	**1.25 (1.06, 1.47)**	**1.23 (1.03, 1.47)**	**1.21 (1.02, 1.44)**
Anxiety symptoms	135 (23.1)	**0.63 (0.52, 0.78)**	**0.64 (0.52, 0.80)**	**0.65 (0.52, 0.79)**
Depressive symptoms	196 (33.6)	**0.79 (0.67, 0.95)**	**0.81 (0.67, 0.97)**	**0.84 (0.70, 0.99)**

Model I: Adjusted for age, sex, place of residence, educational stage, family economic status, and parental educational level. Model II was further adjusted for sleep duration, social media time, sedentary time, physical activity time, parental healthy lifestyle habits, parental attention to dietary balance, and body type. Bold values indicated statistical significance *p* < 0.05.

Restricted cubic spline analyses showed that a higher dietary frequency score was associated with progressively higher odds of higher body satisfaction (*p* for overall = 0.007, *p* for nonlinear = 0.491). In contrast, increasing dietary frequency scores were associated with lower odds of anxiety symptoms (*p* for overall < 0.001, *p* for nonlinear = 0.619) and depressive symptoms (*p* for overall = 0.014, *p* for nonlinear = 0.972) (Figure 1).

**Figure 1:**
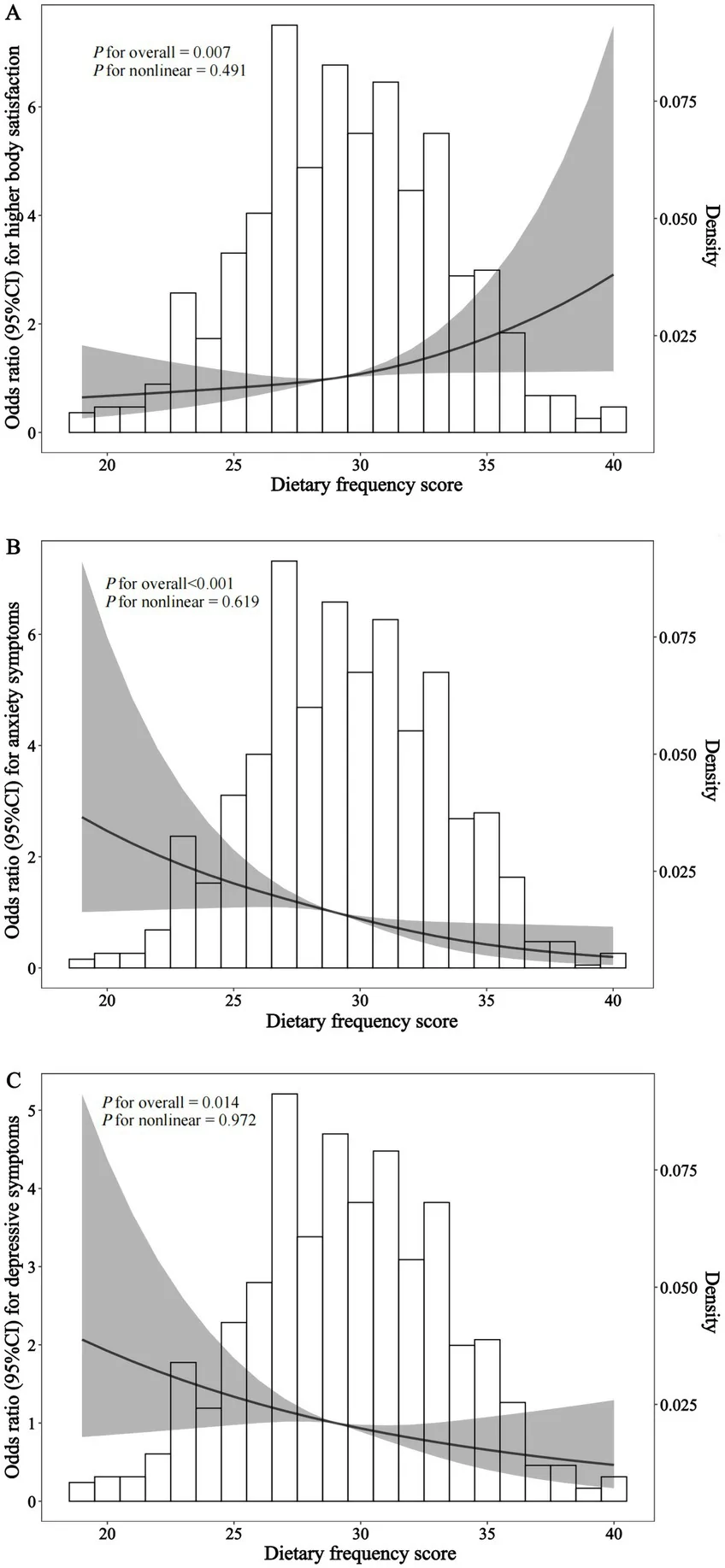
Restricted Cubic Spline for the associations of dietary frequency score with higher body satisfaction, anxiety symptoms, and depressive symptoms. **(A–C)** Show the restricted cubic spline associations of the dietary frequency score with **(A)** higher body satisfaction, **(B)** anxiety symptoms, and **(C)** depressive symptoms, respectively. Three knots were placed at the 10th, 50th, and 90th percentiles of the dietary frequency score distribution, corresponding to scores of 25, 29, and 34, respectively. The median score of 29 was used as the reference value. Solid lines represent adjusted odds ratios, shaded areas represent 95% confidence intervals, and histograms show the distribution of the dietary frequency score. Models were adjusted for age, sex, educational stage, place of residence, family economic status, parental educational level, sleep duration, social-media use time, sedentary time, physical-activity time, parental healthy lifestyle habits, parental attention to dietary balance, and body type.

### 3.3 Stratified analyses

Exploratory stratified analyses showed generally consistent directions of association across subgroups defined by age, sex, social media time, sleep duration, physical activity time, sedentary time, and body type (Figure 2). After Benjamini–Hochberg FDR correction for the interaction tests, none of the interaction terms was statistically significant, providing no clear evidence of effect modification across the examined subgroups.

**Figure 2:**
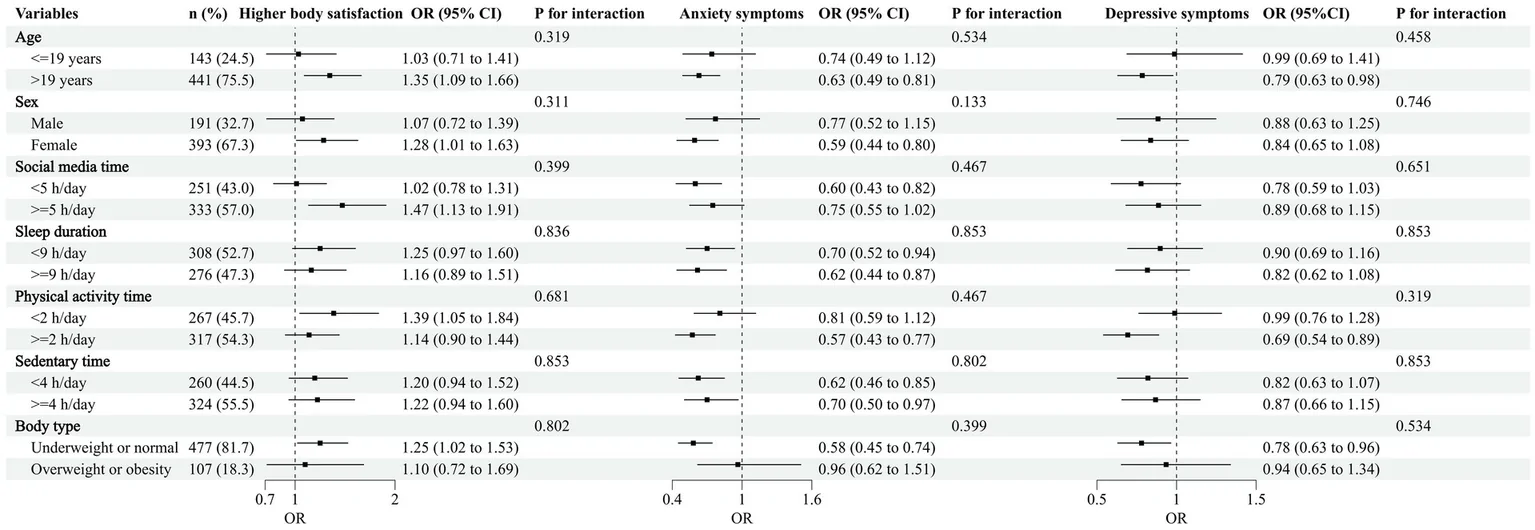
Stratified associations of dietary frequency score with higher body satisfaction, anxiety symptoms, and depressive symptoms. Age was stratified at 19 years according to the WHO definition of adolescence. Social media time, sleep duration, physical activity time, and sedentary time were grouped according to median. *p* values for interaction were obtained from multiplicative interaction terms between the dietary frequency score and each stratification variable and were adjusted across all interaction tests using the Benjamini–Hochberg false discovery rate procedure. Models were adjusted for age, sex, place of residence, educational stage, family economic status, parental educational level, sleep duration, social media time, sedentary time, physical activity time, parental healthy lifestyle habits, parental attention to dietary balance, and body type, except for the corresponding stratification variable.

### 3.4 Counterfactual mediation analysis

Counterfactual mediation analyses identified small statistical indirect associations through higher body satisfaction (Figure 3; Table 3). For anxiety symptoms, the total effect of a 1-SD increase in the dietary frequency score was OR = 0.63 (95% CI: 0.50–0.76), comprising a natural direct effect of OR = 0.65 (95% CI: 0.52–0.79) and a natural indirect effect through higher body satisfaction of OR = 0.97 (95% CI: 0.93–0.99). The indirect component accounted for 6.8% of the total association. For depressive symptoms, the total effect was OR = 0.81 (95% CI: 0.67–0.96), with a natural direct effect of OR = 0.84 (95% CI: 0.70–0.99) and a natural indirect effect of OR = 0.96 (95% CI: 0.92–0.99), corresponding to a mediation proportion of 17.3%.

**Figure 3:**
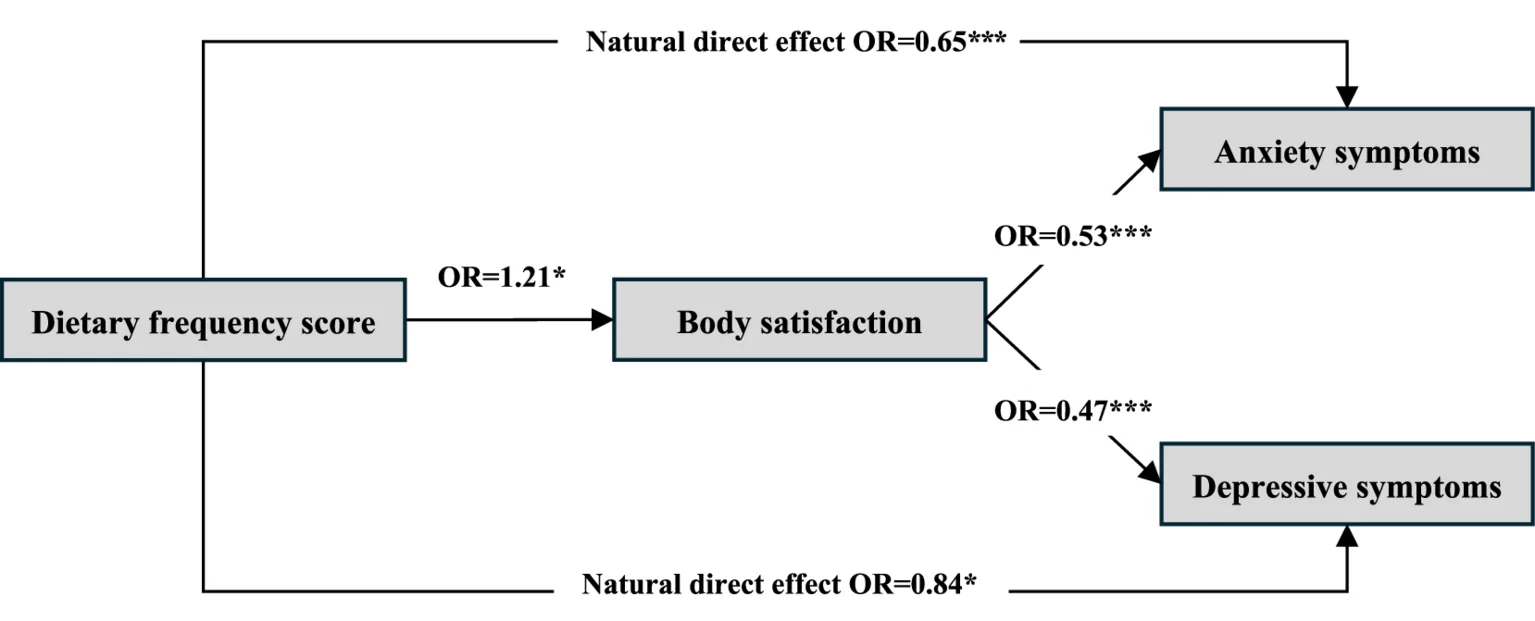
Mediating role of body satisfaction in the associations between dietary frequency score and anxiety and depressive symptoms. *p < 0.05; ***p < 0.001. Models were adjusted for age, sex, place of residence, educational stage, family economic status, parental educational level, sleep duration, social media time, sedentary time, physical activity time, parental healthy lifestyle habits, parental attention to dietary balance, and body type.

**Table 3:** Counterfactual mediation analysis.

Path	OR	95% CI	Mediation proportion	Mediator-model Nagelkerke (*R*^2^)	Outcome-model Nagelkerke (*R*^2^)
Outcome: anxiety symptoms				0.039	0.085
Total effect	0.63	(0.50, 0.76)	—		
Natural direct effect: Dietary frequency score → Anxiety symptoms	0.65	(0.52, 0.79)	—		
Natural indirect effect: Dietary frequency score → Higher body satisfaction → Anxiety symptoms	0.97	(0.93, 0.99)	6.8%		
Outcome: depressive symptoms				0.039	0.068
Total effect	0.81	(0.67, 0.96)	—		
Natural direct effect: Dietary frequency score → Depressive symptoms	0.84	(0.70, 0.99)	—		
Natural indirect effect: Dietary frequency score → Higher body satisfaction → Depressive symptoms	0.96	(0.92, 0.99)	17.3%		

The dietary frequency score was standardized before mediation analysis; therefore, the reported ORs correspond to a 1-SD increase in the dietary frequency score. Nagelkerke’s pseudo-*R*^2^ was reported for both the mediator and outcome logistic regression models. Confidence intervals were obtained from 5,000 bootstrap resamples. Models were adjusted for age, sex, educational stage, place of residence, family economic status, parental educational level, sleep duration, social media time, sedentary time, physical activity time, parental healthy lifestyle habits, parental attention to dietary balance, and body type.

### 3.5 Sensitivity analyses

To assess the robustness of the primary findings with respect to recruitment-site imbalance, individual dietary components, and potential reverse associations, several sensitivity analyses were performed. First, in the single-hospital analysis, the associations of the dietary frequency score with higher body satisfaction, anxiety symptoms, and depressive symptoms remained consistent with the primary analyses (Supplementary Table S2). Second, food-specific analyses showed that only selected food groups were associated with the study outcomes, with relatively stronger positive associations observed for higher consumption frequencies of dairy products, fish, and fruit with higher body satisfaction; stronger inverse associations of higher consumption frequencies of meat, fish, vegetables, and fruit and lower consumption frequency of fried foods with anxiety symptoms; and stronger inverse associations of higher consumption frequencies of fruit, meat, and fish with depressive symptoms. (Supplementary Table S3). Third, reverse-direction mediation analyses indicated that reverse or bidirectional relationships could not be excluded (Supplementary Table S4).

## 4 Discussion

### 4.1 Main findings

This study examined the associations of dietary frequency with body satisfaction, anxiety symptoms, and depressive symptoms among offspring of healthcare workers recruited in Zhejiang Province. Several main findings emerged. First, a higher dietary frequency score was associated with higher body satisfaction and lower odds of anxiety and depressive symptoms after adjustment for demographic, lifestyle, and family-related covariates. Second, restricted cubic spline analyses indicated approximately linear associations of the dietary frequency score with all three outcomes. Third, counterfactual mediation analyses identified small statistical indirect associations through higher body satisfaction, accounting for 6.8% of the association with anxiety symptoms and 17.3% of that with depressive symptoms.

### 4.2 Comparison with other studies

Our finding that a higher dietary frequency score was associated with higher body satisfaction adds to the growing evidence linking overall dietary behaviors with body-image evaluation. Its emphasis on more frequent consumption of nutritionally favorable foods and less frequent consumption of sugary and fried foods is broadly consistent with features of healthy dietary patterns such as the Mediterranean diet. In a representative study of 761 Spanish adolescents (42), those with body satisfaction showed higher adherence to the Mediterranean diet, and Mediterranean-diet adherence was among the lifestyle factors associated with body satisfaction. However, this relationship has not been consistently observed. Another study of Spanish adolescents (43) found no significant association between Mediterranean-diet adherence and body satisfaction, while a systematic review of 30 adolescent studies (12) reported considerable heterogeneity in the associations between dietary habits and body image satisfaction. Intervention studies have also provided some support for a relationship between dietary behaviors and body image, as a systematic review of nutrition-education interventions among Asian young people (44) found that most interventions improved unhealthy food intake and body image misperception, although these interventions were generally multicomponent and could not isolate the effect of dietary change alone. Our food-specific analyses provide further context, showing relatively stronger positive associations of dairy products, fish, and fruit with higher body satisfaction. Previous studies have similarly linked greater fruit and vegetable intake with more favorable body image evaluations (45, 46), although null findings have also been reported (47). These inconsistencies may reflect differences in age, developmental stage, weight status, sociocultural context, body image measures, and covariate adjustment. The associations with fish and dairy products may additionally reflect aspects of nutritional adequacy and physical development, as both provide high-quality protein and nutrients relevant to growth and body composition (48).

Higher dietary frequency scores were associated with lower odds of both anxiety and depressive symptoms, broadly consistent with studies of overall dietary patterns in young populations (49, 50). A recent systematic review found that greater adherence to the Mediterranean diet was generally associated with better mental health in children and adolescents, although the evidence was more consistent for depressive symptoms than for anxiety (49). Similarly, among 698 Spanish adolescents, moderate and high adherence to the Mediterranean diet were associated with substantially lower odds of depressive symptoms, whereas comparable associations were not clearly observed for anxiety (50). These findings suggest that the relationship between diet and emotional health may vary according to the psychological outcome examined. Evidence from individual foods also appears less consistent. In a longitudinal study of 13,887 Canadian adolescents (11), higher sugar-sweetened beverage consumption predicted more severe depressive and anxiety symptoms 1 year later, whereas greater fruit and vegetable consumption was associated with better psychological well-being but not with lower anxiety or depressive symptoms. Our food-specific analyses reflected a similarly nonuniform pattern. Higher consumption of meat, fish, vegetables, and fruit and lower consumption of fried foods were more strongly associated with lower odds of anxiety symptoms, whereas higher consumption of fruit, meat, and fish showed stronger inverse associations with depressive symptoms. For depression, this pattern partly accords with previous reviews identifying fruit, vegetables, and fish among the foods most consistently associated with fewer depressive symptoms in youth, although findings across individual food groups remain heterogeneous (51, 52). Prospective evidence has also been inconsistent; in a community-based cohort, Mediterranean-diet adherence, fruit and vegetable intake, and fish intake were no longer associated with subsequent depressive symptoms after adjustment for behavioral and psychosocial factors (53). Evidence linking individual foods specifically to anxiety is even more limited, making our anxiety-related findings more difficult to interpret in food-specific terms. The association observed for meat also warrants caution because our dietary assessment did not distinguish lean, red, and processed meat, which differ substantially in nutritional composition (54). Accordingly, these food-specific findings add nuance to the observed diet–mental health associations and highlight variation across individual foods.

The counterfactual mediation analyses identified indirect associations involving body satisfaction for both anxiety and depressive symptoms, with the estimated indirect component being larger for depression than for anxiety (17.3% vs. 6.8%). This is consistent with longitudinal evidence showing that body satisfaction is associated with later depressive symptoms and anxiety trajectories during adolescence and young adulthood (6, 7). In the UK Millennium Cohort Study, lower body satisfaction at age 11 was associated with more depressive symptoms at age 14, and accounted statistically for part of the association between childhood BMI and later depression (55). Similarly, a 10-year longitudinal analysis from Project EAT found prospective associations between body satisfaction and depressive symptoms from adolescence into early adulthood, although the dominant direction differed by sex and developmental stage (6). By comparison, evidence for anxiety appears more symptom-specific. Body satisfaction has been associated prospectively with social anxiety and panic symptoms, but less consistently with generalized or separation anxiety trajectories (7). Body satisfaction may therefore represent an important psychosocial dimension connecting everyday dietary behaviors with emotional well-being. Less favorable body evaluations are closely associated with negative self-evaluation, appearance-related shame, and lower self-esteem, as well as with dieting, restrictive eating, emotional eating, and unhealthy weight-control practices (56, 57). These interrelated psychological and behavioral features provide a plausible context for the indirect associations observed in our study and may also help explain why the estimated indirect component was larger for depression than for anxiety. At the same time, the remaining direct associations indicate that body satisfaction captures only part of the relationship between dietary frequency and emotional symptoms. Given the concurrent measurement of these variables, the indirect estimates are interpreted as statistical associations rather than as evidence of temporal mediation.

### 4.3 Potential mechanisms

Several mechanisms may underlie the observed associations. First, a more favorable dietary-frequency profile may reflect greater intake of nutrients involved in neurotransmitter synthesis, neuronal membrane function, neuroplasticity, and emotional regulation, including folate, vitamins B6 and B12, iron, zinc, and long-chain omega-3 fatty acids (58, 59). Second, dietary composition may influence anxiety and depressive symptoms through systemic inflammatory and oxidative pathways, as well as through changes in gut microbial composition and metabolite production along the microbiota–gut–brain axis (59, 60). Third, psychosocial mechanisms may involve body-image evaluation. Lower body satisfaction is associated with lower self-esteem and greater emotional distress (61), while body-image concerns may also influence restrictive eating and other weight-control behaviors (12), thereby linking body evaluation with everyday dietary practices and psychological health.

### 4.4 Limitations

Several limitations should be considered. First, the cross-sectional design and concurrent assessment of dietary frequency, body satisfaction, and psychological symptoms preclude determination of temporal ordering. Reverse and bidirectional associations remain possible, and the estimated indirect effects should therefore be interpreted as statistical indirect associations rather than evidence of causal mediation. Second, dietary intake and psychological measures were self-reported and may be affected by recall or reporting bias. The dietary frequency score captured consumption frequency but not portion size, total energy or nutrient intake, degree of food processing, or cooking methods; consequently, it cannot characterize nutritional exposure with the precision of quantitative dietary assessment. Third, although the models incorporated a broad range of demographic, lifestyle, parental, and age-appropriate body-type covariates, residual confounding may remain. Potentially relevant factors not assessed included pubertal status, chronic health conditions, family structure, dieting and weight-control behaviors, eating-disorder symptoms, family psychiatric history, peer and media influences, and more detailed socioeconomic indicators such as household income and parental occupation. Moreover, the generally modest effect estimates and low Nagelkerke pseudo-*R*^2^ values indicated limited model-level explanatory performance. Accordingly, the observed associations should not be interpreted as demonstrating clinically meaningful effects or as suggesting that changes in the consumption frequency of these selected foods alone would substantially improve psychological health. Fourth, participants were offspring of healthcare workers recruited through four hospitals in Zhejiang Province. They may differ from other Chinese young people in parental health literacy, socioeconomic circumstances, access to health-related resources, and family health practices. Therefore, the findings should not be generalized to all children, adolescents, or young adults in China. Recruitment was also highly concentrated in one hospital, and the unequal distribution across hospitals may have introduced site-related selection or within-site correlation. Although the single-hospital sensitivity analysis yielded broadly similar estimates, hospital-level clustering could not be fully quantified. Finally, family identifiers and sibling status were unavailable, so the possibility that some participants belonged to the same family, and therefore shared unmeasured familial characteristics, could not be excluded.

## 5 Conclusion

Higher dietary frequency scores were associated with greater body satisfaction and lower odds of anxiety and depressive symptoms among offspring of healthcare workers recruited in Zhejiang Province. Food-specific analyses further indicated that the magnitude of these associations varied across individual food groups. Counterfactual mediation analyses also identified indirect associations via body satisfaction for both anxiety and depressive symptoms, with a larger estimated indirect component for depressive symptoms. These findings suggest that body satisfaction may represent an important psychosocial dimension in the association between dietary behaviors and emotional health. From a prevention perspective, strategies targeting anxiety and depressive symptoms in young people may benefit from combining the promotion of favorable dietary behaviors with attention to body satisfaction, thereby supporting a more integrated approach to mental health promotion and prevention.

## Data Availability

The raw data supporting the conclusions of this article will be made available by the authors, without undue reservation.
