# Supplementary material for: Dietary frequency, body satisfaction, anxiety symptoms, and depressive symptoms among offspring of healthcare workers in China
Source: Front Nutr. 2026 Oct 15;13:1908874. doi: 10.3389/fnut.2026.1908874 (PMC13716655; doi:10.3389/fnut.2026.1908874)
Supplement: Supplementary file 1 [file Supplementary_file_1.docx]

**Supplementary Fig. 1: Flow chart of the study population**

**Participants were recruited at four hospitals between July and September 2025, *n*=609**

• First Affiliated Hospital: *n*=487

• Sir Run Run Shaw Hospital: *n*=49

• School and Hospital of Stomatology: *n*=37

• Wenzhou People's Hospital: *n*=36

**Exclude those participants who aged <10 or >24, *n*=24**

**Exclude those participants who did not respond, *n*=1**

**The remaining participants were included in the study, *n*=584**

**Supplementary Table 1: Multicollinearity Assessment (GVIFs)**

| **Variable** | **df** | **GVIF** | **GVIF^(1/(2*Df))** |
| --- | --- | --- | --- |
| Dietary frequency score | 1 | 1.150 | 1.073 |
| Sex | 1 | 1.131 | 1.064 |
| Age | 1 | 3.699 | 1.923 |
| Educational stage | 1 | 3.702 | 1.924 |
| Place of residence | 2 | 1.420 | 1.092 |
| Parental education | 2 | 1.558 | 1.117 |
| Family economic status | 3 | 1.374 | 1.054 |
| Sleep duration | 1 | 1.154 | 1.074 |
| Social media use time | 1 | 1.858 | 1.363 |
| Sedentary time | 1 | 1.494 | 1.222 |
| Physical activity time | 1 | 1.044 | 1.022 |
| Parental healthy lifestyle habits | 3 | 1.338 | 1.050 |
| Parental attention to dietary balance | 2 | 1.234 | 1.054 |
| Body type | 3 | 1.220 | 1.034 |

**Supplementary Table 2: Single-hospital sensitivity analysis of the associations of dietary frequency score with body satisfaction, anxiety symptoms, and depressive symptoms**

| **Outcome** | ***n* (%)** | **Dietary** **frequency score, *OR* (95% CI)** | | |
| --- | --- | --- | --- | --- |
|  |  | **Crude** | **Model I** | **Model II** |
| **Higher body satisfaction** | 238 (49.0) | **1.08 (1.03–1.14)** | **1.09 (1.03–1.14)** | **1.08 (1.02–1.15)** |
| **Anxiety symptoms** | 118 (24.3) | **0.87 (0.82–0.92)** | **0.88 (0.83–0.94)** | **0.88 (0.83–0.95)** |
| **Depressive symptoms** | 171 (35.2) | **0.92 (0.88–0.97)** | **0.94 (0.89–0.99)** | 0.95 (0.89–1.00) |

Note: The study population was restricted to offspring of healthcare workers from the First Affiliated Hospital of Wenzhou Medical University (*n*=486).

Among the 487 participants recruited through the First Affiliated Hospital of Wenzhou Medical University, one participant was excluded because their age fell outside the predefined eligibility range, leaving 486 participants for analysis.

Model I: Adjusted for age, sex, place of residence, educational stage, family economic status, and parental educational level. Model II was further adjusted for sleep duration, social media time, sedentary time, physical activity time, parental healthy lifestyle habits, parental attention to dietary balance, and body type. Bold values indicated statistical significance *P* <0.05.

**Supplementary Table 3: Associations of individual food-frequency components with body satisfaction, anxiety symptoms, and depressive symptoms**

| **Food group** | **OR (95% CI)** | | | | | |
| --- | --- | --- | --- | --- | --- | --- |
|  | **Higher body satisfaction** | ***P*** | **Anxiety symptoms** | ***P*** | **Depressive symptoms** | ***P*** |
| Meat | 1.08 (0.87–1.34) | 0.498 | **0.63 (0.49–0.80)** | **<0.001** | **0.78 (0.62–0.98)** | **0.035** |
| Fish | **1.29 (1.07–1.55)** | **0.006** | **0.76 (0.61–0.95)** | **0.015** | **0.78 (0.64–0.95)** | **0.012** |
| Vegetables | 1.01 (0.82–1.26) | 0.893 | **0.61 (0.48–0.77)** | **<0.001** | 0.86 (0.69–1.07) | 0.181 |
| Fruit | **1.27 (1.05–1.54)** | **0.012** | **0.71 (0.57–0.88)** | **0.002** | **0.77 (0.63–0.94)** | **0.009** |
| Eggs | 1.08 (0.88–1.31) | 0.461 | 0.80 (0.63–1.00) | 0.053 | 1.01 (0.82–1.25) | 0.897 |
| Soy products | 1.09 (0.89–1.32) | 0.401 | 0.98 (0.78–1.24) | 0.880 | 0.98 (0.80–1.21) | 0.884 |
| Pickled foods | 1.02 (0.83–1.26) | 0.829 | 0.82 (0.64–1.05) | 0.116 | 0.90 (0.72–1.12) | 0.337 |
| Sugary foods/drinks | 1.01 (0.83–1.23) | 0.929 | 0.85 (0.67–1.09) | 0.199 | 0.95 (0.77–1.18) | 0.663 |
| Fried foods | 1.05 (0.83–1.32) | 0.705 | **0.75 (0.57–0.98)** | **0.036** | 0.92 (0.72–1.18) | 0.523 |
| Dairy products | **1.33 (1.10–1.61)** | **0.003** | 0.83 (0.67–1.03) | 0.096 | 0.84 (0.69–1.02) | 0.083 |

Adjusted for age, sex, place of residence, educational stage, family economic status, parental educational level, sleep duration, social media time, sedentary time, physical activity time, parental healthy lifestyle habits, parental attention to dietary balance, and body type. Bold values indicated statistical significance *P* <0.05.

Scores for pickled foods, sugary foods/drinks, and fried foods were reverse-coded, with higher scores indicating a more favorable dietary-frequency profile

**Supplementary Table 4:** **Reverse-direction mediation sensitivity analysis**

| **Exposure** | **Effect** | **Standardized estimate** | **95% CI** | **Mediation proportion** | **Mediator model Nagelkerke (R^2^)** | **Outcome model (R^2^)** |
| --- | --- | --- | --- | --- | --- | --- |
| **Anxiety symptoms** | Direct effect | −0.412 | −0.596 to −0.225 | — | 0.052 | 0.061 |
|  | Indirect effect | −0.025 | −0.062 to 0.001 | 5.7% |  |  |
|  | Total effect | −0.437 | −0.615 to −0.252 | — |  |  |
| **Depressive symptoms** | Direct effect | −0.177 | −0.348 to −0.007 | — | 0.068 | 0.039 |
|  | Indirect effect | −0.032 | −0.072 to −0.001 | 15.3% |  |  |
|  | Total effect | −0.209 | −0.379 to −0.041 | — |  |  |

Notes: Reverse-direction counterfactual mediation analyses were adjusted for sex, age, place of residence, family economic status, parental educational level, sleep duration, social media time, sedentary time, physical activity time, parental healthy lifestyle habits, parental attention to dietary balance, and body type.

Higher body satisfaction was modeled as a binary mediator using logistic regression, whereas the standardized dietary frequency score was modeled as a continuous outcome using linear regression.

Standardized estimates are expressed in SD units of the dietary frequency score.

The mediator-model R^2^ represents Nagelkerke’s pseudo-R^2^, whereas the outcome-model R^2^ represents the conventional coefficient of determination. Confidence intervals were obtained from 5,000 bootstrap resamples.
